# An MRI-guided HIFU-triggered wax-coated capsule for supertargeted drug release: a proof-of-concept study

**DOI:** 10.1186/s41747-019-0090-9

**Published:** 2019-03-05

**Authors:** Simon Matoori, Maurizio Roveri, Peter Tiefenboeck, Annatina Romagna, Olha Wuerthinger, Orpheus Kolokythas, Johannes M. Froehlich

**Affiliations:** 1 Institute of Pharmaceutical Sciences, Department of Chemistry and Applied Biosciences, ETH Zurich, Vladimir-Prelog-Weg 1-5/10, 8093 Zurich, Switzerland; 2 Clinical Research Group, Klus Apotheke Zurich, Zurich, Switzerland; 3 Department of Radiology, Kantonsspital Winterthur, Winterthur, Switzerland; 4 Department of Radiology, University of Washington Medical Center, Seattle, WA USA

**Keywords:** Contrast media, Drug delivery systems, Gastrointestinal tract, High-intensity focused ultrasound, Magnetic resonance imaging

## Abstract

**Background:**

Externally controlling and monitoring drug release at a desired time and location is currently lacking in the gastrointestinal tract. The aim of the study was to develop a thermoresponsive wax-coated capsule and to trigger its release upon applying a magnetic resonance imaging (MRI)-guided high-intensity focused ultrasound (HIFU) pulse.

**Methods:**

Capsules containing a lyophilised gadolinium-based contrast agent (GBCA) were coated with a 1:1 (mass/mass) mixture of lanolin and cetyl alcohol (melting point ≈43 °C) and exposed to simulated gastric and intestinal fluids (United States Pharmacopoeia) at 37 °C for 2 and 24 h, respectively. In a HIFU gel phantom, wax-coated capsules (*n* = 3) were tracked based on their T1- and T2-hypointensity by 1.5-T T1- and T2-weighted MRI pre- and post-exposure to an MRI-guided HIFU pulse.

**Results:**

Lanolin/cetyl alcohol-coated capsules showed high resistance to simulated gastrointestinal fluids. In a gel phantom, an MRI-guided HIFU pulse punctured the wax coating, resulting in the hydration and release of the encapsulated lyophilised GBCA and yielding a T1-hyperintense signal close to the wax-coated capsule.

**Conclusion:**

We provide the proof-of-concept of applying a non-invasive MRI-guided HIFU pulse to actively induce the disintegration of the wax-coated capsule, and a method to monitor the release of the cargo via T1-weighted MRI based on the hydration of an encapsulated lyophilised GBCA. The wax-coated capsule platform enables temporally and spatially supertargeted drug release via the oral route and promises to address a currently unmet clinical need for personalised local therapy in gastrointestinal diseases such as inflammatory bowel diseases and cancer.

**Electronic supplementary material:**

The online version of this article (10.1186/s41747-019-0090-9) contains supplementary material, which is available to authorized users.

## Key points

- A wax coating (melting point 43 °C) conferred thermoresponsive properties to capsules (*n* = 6).
- T1- and T2-weighted MRI allowed real-time tracking of wax-coated capsules (*n* = 3).
- An MRI-guided high-intensity focused ultrasound pulse triggered the release of the cargo (*n* = 3).
- A lyophilised contrast agent allowed monitoring the release on T1-weighted MRI (*n* = 3).
- Wax-coated capsules may provide a platform for personalised local therapy in gastroenterology.

## Background

In certain diseases, drugs are administered systemically even though their therapeutic action is only required at a specific location [1]. In patients suffering from non-metastatic solid tumours (*e.g.*, gastrointestinal (GI) tumours, breast cancer), chemotherapeutics are often applied systemically despite the confinement of the tumour to a restricted area, which may negatively influence the benefit-risk ratio of perioperative or adjuvant chemotherapy [2, 3]. Preferably, the drug would be released at the site of the lesion and at a freely selectable time via an external trigger. Such a delivery system promises to improve efficacy and safety by reaching high drug concentrations in the proximity of the lesion while sparing the healthy tissue from potentially noxious drug exposure.

Even though controlled release formulations relying on time-dependent or stimuli-responsive release are widespread, the possibility of externally controlling and monitoring the drug release at a desired time and location (*i.e.* supertargeted drug release) is currently lacking in the GI tract [4–7]. Thermoresponsive drug delivery systems, whose release is triggered by a local temperature increase, are a promising method to enable externally controlled drug release. Even though a variety of thermoresponsive systems have been described (*e.g.* hydrogels, metal or polymeric nanoparticles, liposomes, micelles), their application in harsh environments such as the GI tract or highly inflamed lesions is generally limited by their stability [8–15]. Furthermore, such systems may be difficult to locate in dynamic body cavities such as the GI tract. In the age of personalised medicine, novel delivery systems are required to address this currently unmet clinical need in local therapy in the GI tract.

To enable a site- and time-specific drug release, it is essential to determine the location of the drug carrier (here a capsule), preferentially in real time [16]. Non-invasive and non-irradiating imaging modalities such as magnetic resonance imaging (MRI) and MRI-based imaging biomarkers (*e.g.* chemical exchange saturation transfer), ultrasound, and fluorescence-based optical imaging provide the opportunity to visualise the drug carrier in real time and are therefore suitable for temporal and spatial supertargeting [17–21]. To release the therapeutic cargo, a localised temperature increase by an externally controllable heat source such as high-intensity focused ultrasound (HIFU) or electrocauterisation is a promising trigger, allowing for a defined and well-controlled temperature increase at a specific location in a non- or minimally invasive fashion [22, 23]. HIFU, for instance, is clinically used for heat-induced tumour ablation with high spatial accuracy and a predefined target temperature [24, 25]. Moreover, it is generally combined with imaging modalities such as MRI or ultrasound in order to determine the exact localisation of the target lesions and to target the HIFU pulse [26–28]. HIFU would therefore be the ideal triggering device for a thermoresponsive drug delivery system to enable on-demand drug release, and entails the potential of synergistic effects of the released drug and thermoablation of undesired (*e.g.* neoplastic) tissue [19, 29]. Upon oral application, an externally triggered thermosensitive drug delivery system could provide the opportunity to release a therapeutic agent at a specific place along the GI tract (*e.g.* at an inflamed or neoplastic lesion). Such a system promises to reach high drug concentrations at the target site and a low exposition of healthy tissue to the drug.

In this study, we aimed at developing a novel thermosensitive capsule-based drug delivery system and triggering the release of its cargo using a highly localised MRI-guided HIFU pulse. An additional goal was to include a component which allows the monitoring of the release of the capsule content.

## Methods

### Materials

In this paragraph, the origin of the chemicals is listed. More information on the choice of the chemicals is provided in the subsequent paragraphs. European Pharmacopoeia-compliant (Additional file 1: Tables S1, S2), GMP-grade lanolin and cetyl alcohol were obtained from Haenseler AG (Herisau, Switzerland). Hydroxypropyl methylcellulose capsules (size 1, 1.94 cm × 0.68 cm) were purchased from Mueller + Krempel AG (Bulach, Switzerland). Pepsin, pancreatin, and hydrochloric acid were bought from Sigma Aldrich (Buchs SG, Switzerland). Potassium dihydrogen phosphate and barium sulphate were obtained from Merck (Zug, Switzerland), and gadoteric acid meglumine (gadoterate, Gd-DOTA, 0.5 mmol/mL, Dotarem®) was from Guerbet AG (Zurich, Switzerland). All substances used in the wax-coated capsule are widely used highly biocompatible pharmaceutical excipients or inert active principles [30].

### Wax mixtures

**Fig. 1:**
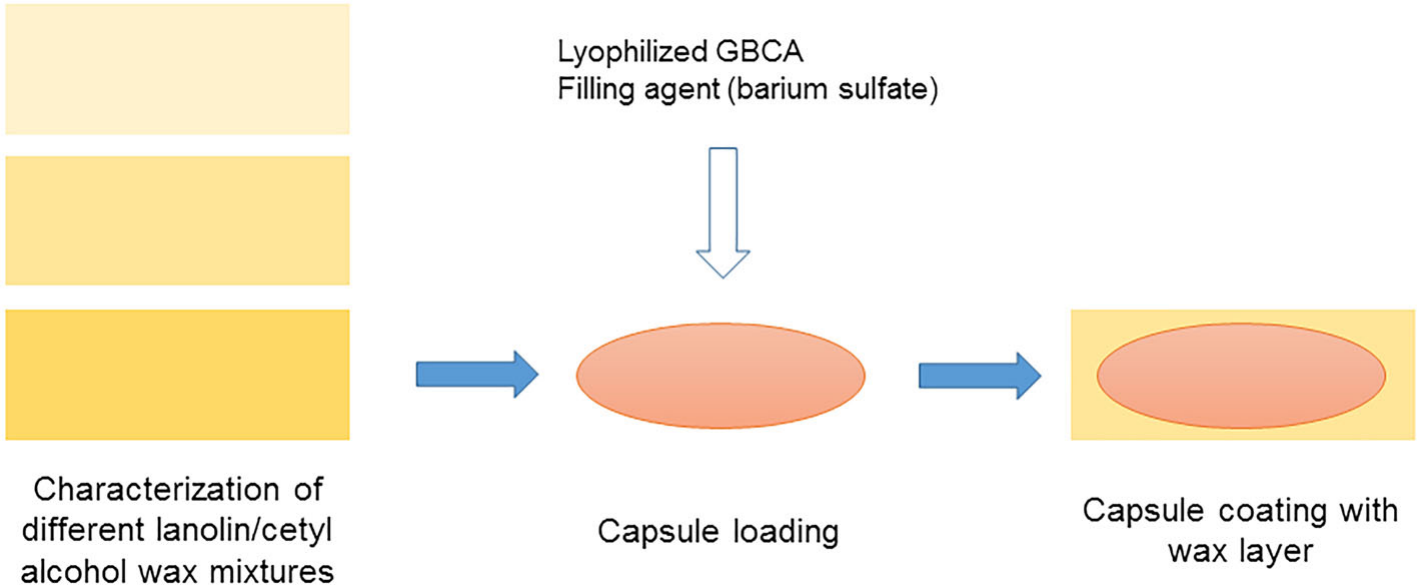
Schematic depiction of the workflow yielding wax-coated capsules. Firstly, different lanolin/cetyl alcohol mixtures are analysed in terms of melting point. Secondly, empty capsules are filled with a lyophilised gadolinium-based contrast agent and a filling agent (barium sulphate). Thirdly, the capsules are coated with the wax mixture (yellow)

Firstly, the melting point of different lanolin/cetyl alcohol mixtures was assessed (Fig. 1). Lanolin and cetyl alcohol were used as a wax coating because of their high biocompatibility, their wide usage as pharmaceutical excipients, and their food excipient status. Lanolin contains mainly (hydroxy-)sterol esters and wax esters, triterpene esters, and small amounts of dihydroxy wax esters, free sterols, free alcohols, and branched fatty acids [31]. To determine the melting point of different lanolin/cetyl alcohol wax mixtures, lanolin and cetyl alcohol were heated to 70 °C in a water bath and mixed in different proportions, and the resultant wax mixtures were added to melting point tubes. Once the wax mixtures were cooled down, a controlled temperature increase in a melting point apparatus (Melting Point B-540, Buchi Labortechnik, Flawil, Switzerland) allowed the precise determination of their melting range.

### Michelangelo method for capsule coating

To coat the capsules, 0.7 g barium sulphate and 0.2 mL gadoteric acid meglumine (0.5 mmol/mL) were mixed, lyophilised (Alpha 2-4 LSC, Christ, Osterode am Harz, Germany) overnight, and subsequently filled into a hydroxypropyl methylcellulose capsule. Barium sulphate was used as a filling agent due to its high density, due to its biocompatibility as it is used as an oral contrast agent for computed tomography (CT) and x-ray, and for its radiopaque properties, which would in addition allow a localisation of the capsule using CT or x-ray (with the downside of using ionising radiation) [32]. As a first step in the capsule coating procedure, a layer of 1 mm wax mixture at approximately 70 °C was poured into a weighing dish (85 × 85 × 24 mm, VWR International, Dietikon, Switzerland). After the wax mixture had cooled down to a viscous state, the filled capsule was placed on the wax layer and another layer of wax mixture at 70 °C was poured into the weighing tray reaching a total height of 2.2 cm. The capsule was carved out from this wax-filled weighing tray using a heated spatula in the following approximate dimensions: 2.2 cm (length), 1.0 cm (height), and 1.1 cm (width). The dimensions of the resultant wax-coated capsules were measured using a calliper.

### Thermoresponsive dissolution test

The release of native and wax-coated hydroxypropyl methylcellulose capsule was investigated using a dissolution apparatus (VK 7000 Dissolution System, VanKel Technology Group, Cary, NC, USA) with a paddle frequency of 250 rotations per minute [33]. Both native and wax-coated capsules contained a lyophilised powder mixture of 0.7 g barium sulphate and 0.2 mL gadoteric acid meglumine (0.5 mmol/mL). The capsules were incubated in 500 mL phosphate buffer 50 mM at pH 6.8 at 37 °C for 30 min and subsequently at 43 °C for 60 min. The turbidity of the aliquots was measured at 400 nm using a plate reader (Infinite 200Pro, Tecan, Maennedorf, Switzerland).

### MRI-based release test

The release of uncoated, gadoterate lyophilisate-containing hydroxypropyl methylcellulose capsules was further assessed using MRI. The capsules were added to a vessel containing 500 mL water at room temperature without mixing, which was placed in a 1.5-T clinical MRI scanner (Achieva, Philips Healthcare, Best, The Netherlands). Static T1-weighted imaging (three-dimensional fast field echo, echo time “shortest”, repetition time 25 ms, flip angle 30°, acquired voxel-size 2.0 × 2.0 × 4.0 mm, total scan duration 56 s) was conducted after 1 and 20 min in order to depict the still intact and disintegrated capsule, respectively. Dynamic T1-weighted imaging (with a series of three-dimensional fast field echo, echo time “shortest”, repetition time 25 ms, flip angle 30°, acquired voxel-size 2.0 × 2.0 × 4.0) was carried out starting from approximately 3.5 min. The release of the capsule content was determined by visual monitoring of T1 intensity.

### Incubation in simulated GI fluids

To test their resistance to simulated body fluids, the wax-coated capsules were incubated in simulated gastric fluid United States Pharmacopoeia (USP, USP 39-NF 34) at pH 1.2 containing 3.2 mg/mL of pepsin (pepsin from porcine gastric mucosa, 3200–4500 units/mg protein) for 2 h and subsequently in simulated intestinal fluid USP at pH 6.8 containing 10 mg/mL of pancreatin for 24 h, both at 37 °C under heavy horizontal shaking at 240 rotations per minute (KS 130 basic, IKA, Staufen, Germany) [34, 35]. Subsequently, the simulated intestinal fluid was decanted and the capsules were washed three times with deionised water. The wax-coated capsules were weighed, and morphologic changes were photographically documented.

### MRI-guided HIFU-induced release test

**Fig. 2:**
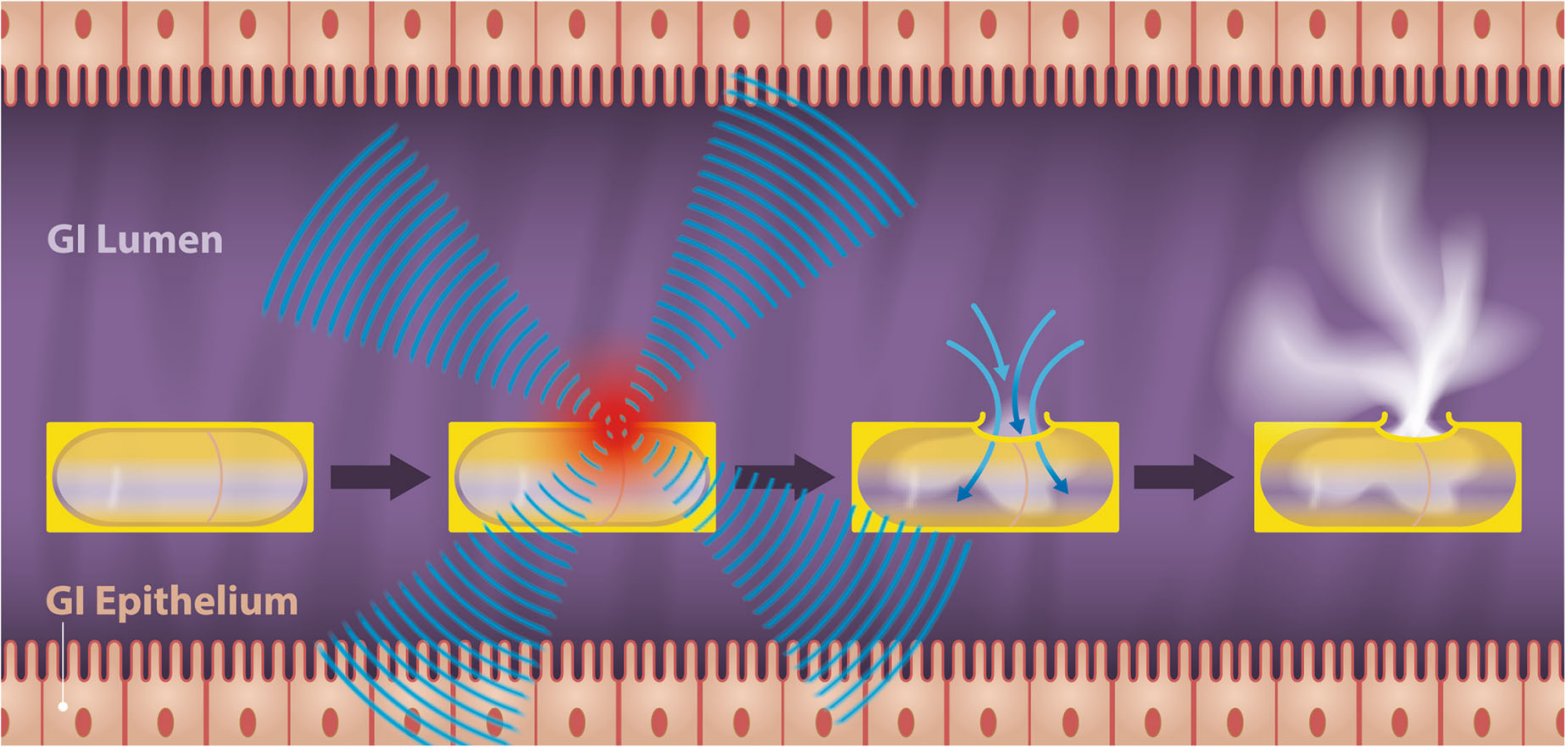
Graphic description of the HIFU-triggered drug release of a wax-coated capsule. Conceptually, once the wax-coated capsule is in the body (*e.g.* in the GI tract), it can be tracked due to its hypointensity on T2-weighted MRI (Fig. 5a). While the wax coating confers resistance to a premature release of the capsule content, its melting point can be designed to be low enough (in this study 43 °C) for a mild non-invasive external heat trigger (*e.g.* by HIFU) to melt a cavity into the coating. Water influx from the gut lumen [59] into the capsule core dissolves the lyophilised GBCA, leading to a well-visible hyperintense signal (white cloud) on T1-weighted MRI. GBCA: Gadolinium-based contrast agent; GI: Gastrointestinal; HIFU: High-intensity focused ultrasound; MRI: Magnetic resonance imaging

To test the MRI-guided HIFU-triggered release of the contrast agent from the wax-coated capsule (Fig. 2), the capsule was placed in an approximately 3-cm-deep cavity of a HIFU gel phantom (Philips Healthcare, Best, The Netherlands) [36, 37]. The cavity was then filled with degassed water and closed with a piece of carved-out gel. The capsule and surrounding structures were imaged on a 1.5-T clinical MRI scanner (Achieva, Philips Healthcare, Best, The Netherlands). The MRI-HIFU temperature mapping and release-triggering experiments were performed using a dedicated MRI-HIFU breast platform (Sonalleve-based prototype, Philips Healthcare, Vantaa, Finland) allowing to perform sonication of the probes inside the magnet [36, 37]. Around the water-filled breast cup containing the gel phantom, there were eight separate 32-element transducers distributed over a 270° circular arc. A T1- (three-dimensional fast field echo, echo time 3.3 ms, repetition time 6.75 ms, flip angle 10°, acquired voxel-size 1.15 × 1.15 × 1.0 mm, turbo field echo factor 36, scan duration 57 s) and a T2-weighted (three-dimensional turbo spin echo, echo time 130 ms, repetition time 1000 ms, turbo spin echo factor 62, acquired voxel-size 1.42 × 1.71 × 1.25 mm, number of signal averages 2, scan duration 182 s) sequence were acquired for morphological visualisation of the capsule outline and subsequent planning of the HIFU triggering experiment.

To plan the triggering experiment, the HIFU beam was localised in the middle of the capsule using the target definition of the HIFU software (8 mm treatment cell; treated volume 0.84 mL) and a test pulse with low acoustic power (30 W, 1195 kHz, 20 s). The triggering experiment was subsequently conducted with a HIFU pulse sequence consisting of an acoustic power of 200 W, a frequency of 1195 kHz, and a duration of 27 s. In parallel, T2 maps of the sample were generated showing the heating stage of the experiment. Approximately 10 min after the HIFU treatment, the capsules were controlled using the same T1- and T2-weighted pulse sequences as described in the first paragraph of the section “MRI-guided HIFU-induced release test” to investigate the release of the gadolinium-based contrast agent (GBCA) from the capsule core. Spatial coherence between the exposed area in HIFU and release area was determined.

### Statistical analysis

To test the significance of the difference in weight pre- and post-incubation (see “Incubation in simulated GI fluids”), we conducted a Mann-Whitney Rank Sum Test using SigmaPlot 13.0 (Systat Software, San Jose, CA, USA).

## Results

### Wax mixture

The melting points of different mixtures of lanolin and cetyl alcohol are listed in Table 1. A mixture of 50% (*m*/*m*) lanolin and 50% (*m*/*m*) cetyl alcohol exhibited the most suitable melting point (approximately 43 °C) as this temperature is only mildly hyperthermic and entails safety margin to pathophysiological and potentially noxious increases in body temperature (*i.e.* hyperthermia, fever reactions; threshold for serum albumin denaturation 50–62 °C) [38, 39].

**Table 1:** Melting ranges of different cetyl alcohol/lanolin mixtures

Cetyl alcohol/lanolin (*m/m*)	Melting point (°C)
100 / 0	49.5–51.1
90 / 10	49.0–49.2
80 / 20	47.9–48.1
70 / 30	46.9–47.2
60 / 40	44.9–45.3
50 / 50	43.0–43.3
40 / 60	40.6–41.1
30 / 70	37.7–38.3
20 / 80	35.7–35.8
10 / 90	35.5–35.6
0 / 100	37.5–38.0

*m/m* mass/mass

### Michelangelo method for capsule coating

**Fig. 3:**
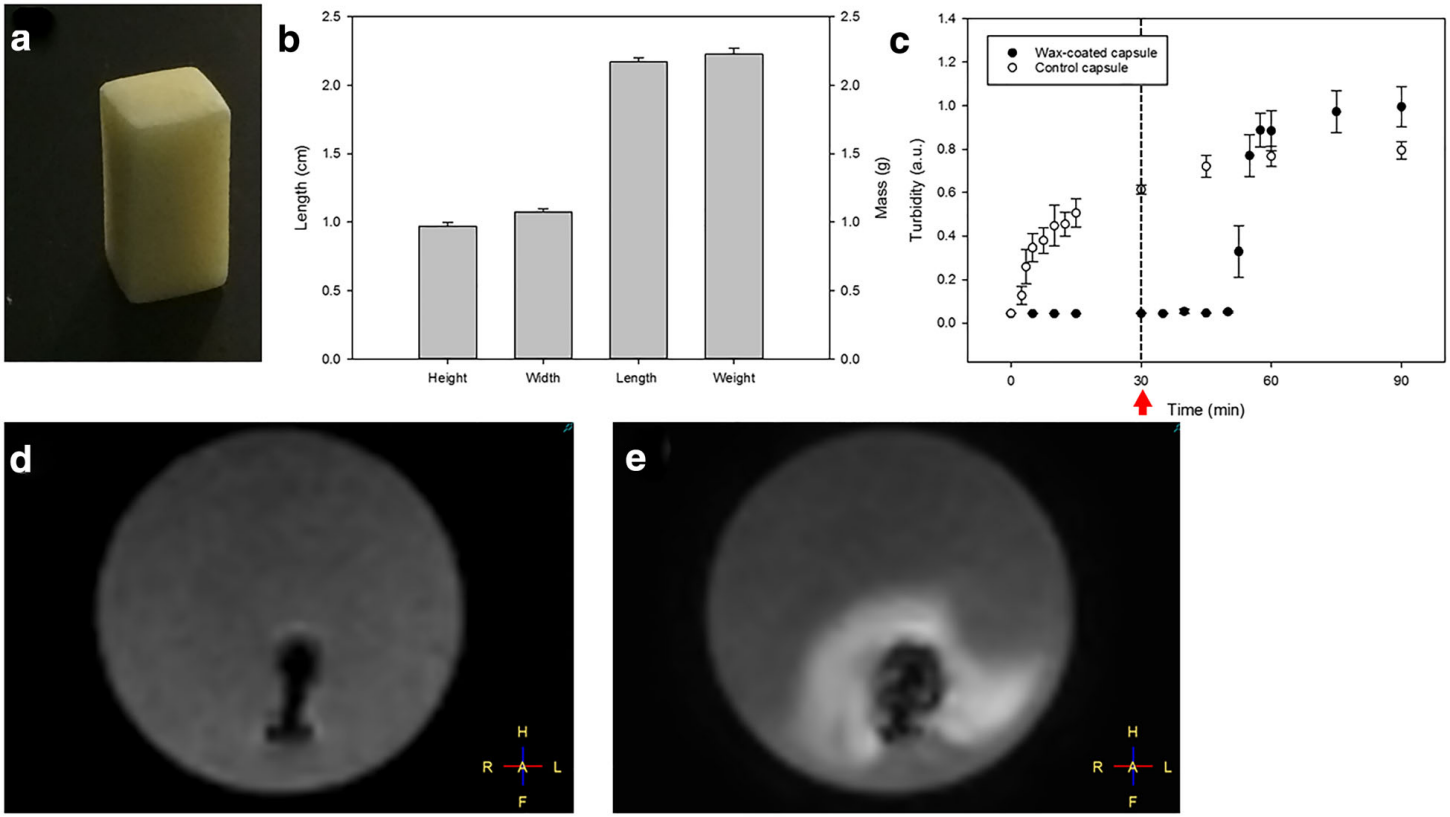
Morphology, dimensions, and release kinetics of lanolin/cetyl alcohol-coated capsules. Wax-coated capsules were made using the Michelangelo method, in which the capsule is carved out of a wax layer, exhibited highly reproducible dimensions and weight (*n* = 6, **a**, **b**). The wax-coated capsules were incubated in phosphate buffer at pH 6.8 for 30 min at 37 °C. The non-coated hydroxypropyl methylcellulose capsules released their content almost immediately while the wax-coated capsules remained intact (*n* = 3, **c**). After 30 min, the temperature was increased to 43 °C, and after a short delay, the wax-coated capsules released their cargo (*n* = 3, **c**). The red arrow indicates the time point when the temperature increase was started. The release kinetics of the uncoated capsules containing freeze-dried gadoterate was further assessed using T1-weighted MRI. One minute after exposing the capsule to water, the capsule was visible as a T1-hypointense signal (*n* = 3, **d**). After 20 min, the hypointense capsule was surrounded by a strong T1-hyperintense signal (*n* = 3, **e**), stemming from the hydration of lyophilised gadoterate after capsule disintegration. All results are shown as mean ± standard deviation

In order to achieve a homogenous wax coating, the capsules were casted in a mixture of 50% (*m*/*m*) lanolin and 50% (*m*/*m*) cetyl alcohol and subsequently carved out in predefined dimensions. The resultant length was 2.17 ± 0.03 cm (mean ± standard deviation), the height 0.97 ± 0.03 cm, the width 1.08 ± 0.02 cm, and the weight 2262 ± 43 mg (*n* = 6, Fig. 3a, b). With a capsule diameter and length of 0.68 cm and 1.94 cm, respectively, the calculated thickness of the coating was 0.15 cm (height), 0.20 cm (width), and 0.12 cm (length).

### Thermoresponsive dissolution test

Upon incubation at pH 6.8 at 37 °C, non-coated hydroxypropyl methylcellulose capsules started releasing their cargo after 2.5 min (*n* = 3, Fig. 3c). The increase in turbidity stemmed from the suspended barium sulphate from the capsule core which has a very low solubility in water (approximately 0.285 mg/100 mL). The wax-coated capsules remained stable during the 30 min incubation at 37 °C and released their cargo only once the temperature had been set to 43 °C (*n* = 3, Fig. 3c).

### MRI-based release test

Exposing the uncoated capsules containing lyophilised gadoterate to water yielded an initially hypointense signal on T1-weighted imaging (*n* = 3, Fig. 3d). After 20 min, a T1-hyperintense signal was clearly visible around the capsule (*n* = 3, Fig. 3e). On dynamic T1-weighted imaging, the release of the capsule content was observed starting from 8.3 ± 0.6 min (*n* = 3, Additional file 2: Movie S1).

### Incubation in simulated GI fluids

**Fig. 4:**
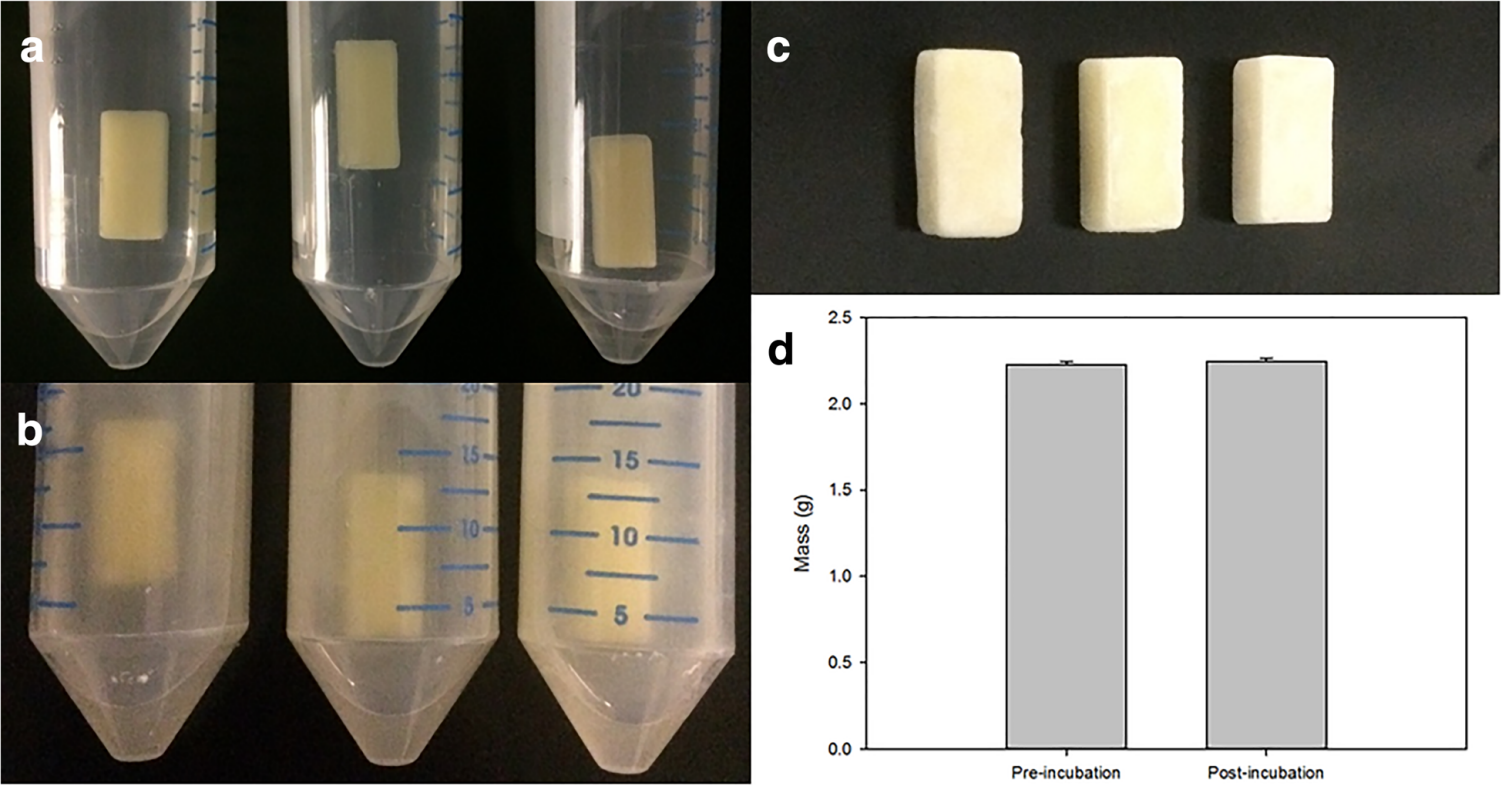
Resistance of wax-coated capsules to simulated GI fluids (*n* = 3). To demonstrate the high resistance of the wax coating to enzymatic activity and low pH, the wax-coated capsules were exposed to simulated GI fluids at 37 °C. Visual control after 2 h incubation in simulated pepsin-containing gastric fluid USP at pH 1.2 (**a**) and subsequent 24 h in pancreatin-containing simulated intestinal fluid USP at pH 6.8 (**b**) revealed no apparent morphological changes (**c**). Moreover, no significant change in mass was observed (*p* = 0.400, mean ± standard deviation, **d**). GI: Gastrointestinal; USP: United States Pharmacopoeia

To test their resistance to body fluids, the wax capsules were incubated in simulated GI fluids at 37 °C (*n* = 3, *i.e.* investigation of three independent replicates, which represents a standard procedure [34]). Incubation in pepsin-containing simulated gastric fluid USP at pH 1.2 for 2 h and subsequently in pancreatin-containing simulated intestinal fluid USP at pH 6.8 for 24 h did not lead to visible morphological changes (Fig. 4a–c). The mass of the three capsules before and after incubation in simulated GI fluids (2.2278 ± 0.0189 g and 2.2474 ± 0.0186 g, respectively) did not significantly differ (*p* = 0.400, Mann-Whitney Rank Sum test, *n* = 3, Fig. 4d).

### MRI-guided HIFU-induced release test

**Fig. 5:**
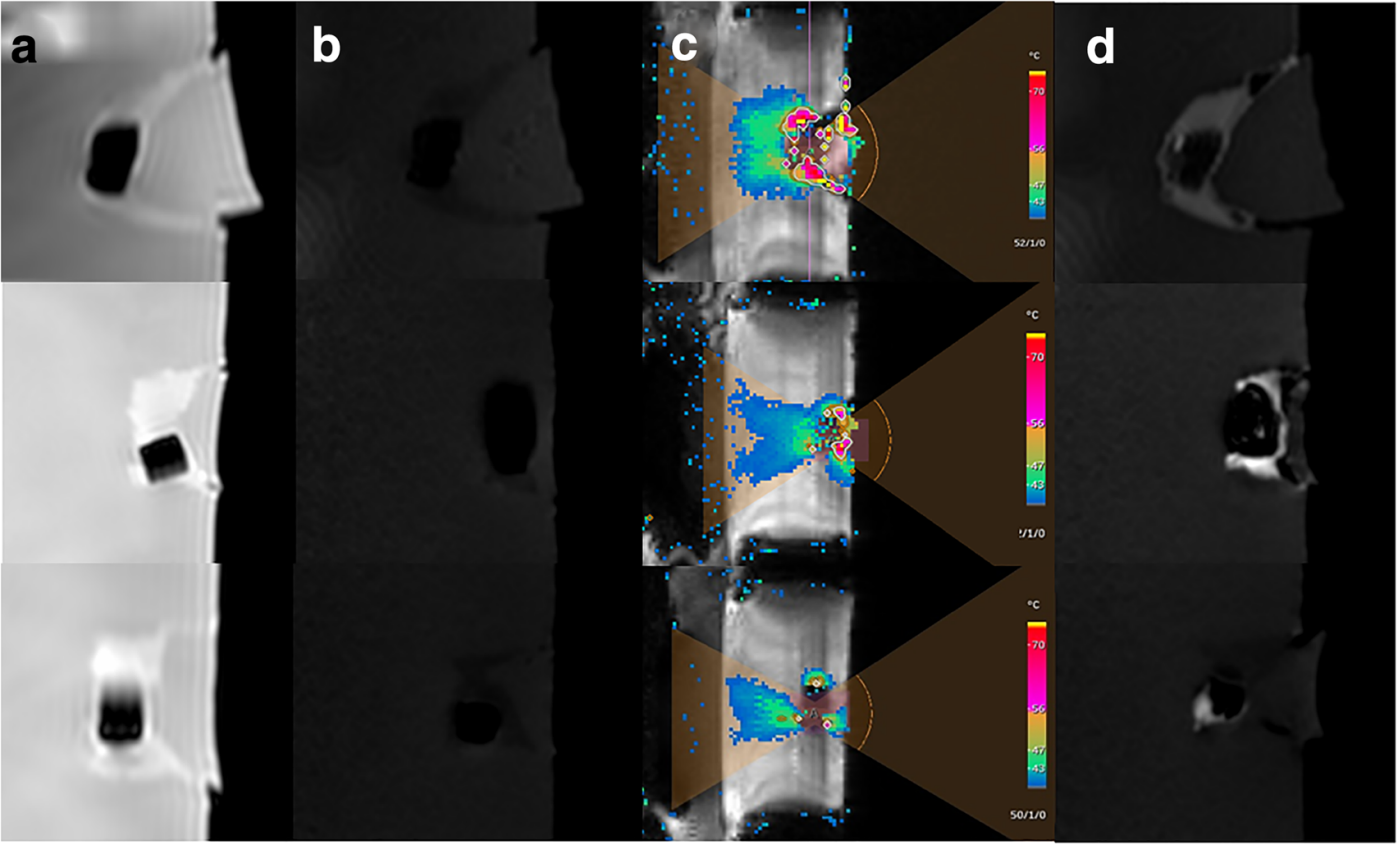
1.5-T MRI-tracked HIFU-triggered release of three wax-coated capsules (*n* = 3, separate experiments shown on three different layers). The position of the wax-coated capsule prior to the application of the HIFU pulse was determined using a 3D T2-weighted turbo spin echo sequence (repetition time 1000 ms, echo time 130 ms, **a**). The intact wax-coated capsule showed no hyperintensity on a 3D T1-weighted fast field echo sequence (repetition time 6.75 ms, echo time 3.3 ms, flip angle 10°), demonstrating that the non-hydrated lyophilised GBCA is not T1-hyperintense (**b**). T2-weighted MRI was used for guidance of the HIFU pulse and MR thermometry (colour mapping reveals thermal exposure, **c**). After the highly localised application of a 200-W HIFU pulse, a distinct T1-hyperintense signal was observed (**d**) using the same sequence as in **b**. The local temperature increase most probably melted the wax coating, allowing water to penetrate the capsule core and hydrate the encapsulated lyophilised GBCA, and thus resulting in the T1-hyperintensity. Hyperintensity varies depending also on the dissolution kinetics of the GBCA in the surrounding water, the amount of surrounding medium, and the imaging delay after HIFU-induced disintegration. GBCA: Gadolinium-based contrast agent; HIFU: High-intensity focused ultrasound

To evaluate the MRI-based tracking and the HIFU-triggered release of the wax-coated capsules, the wax-coated capsule was placed in a HIFU phantom (*n* = 3). Prior to applying the HIFU pulse, a T2-weighted MRI sequence showed the position of the T2-hypointense wax-coated capsule in the HIFU phantom (Fig. 5a). On T1-weighted MRI, no hyperintense signal was observed, demonstrating that the wax-coated capsule was intact and that the GBCA in the lyophilised state was not T1-hyperintense (Fig. 5b). After applying a targeted HIFU pulse with a power of 200 W and a frequency of 1195 kHz close to the lateral side of the capsule (Fig. 5c), a T1-hyperintense signal close to the wax-coated capsule was visible (Fig. 5d). There was a clear matching of the maximum thermal dose location with the T1 enhancement pattern. Most probably, the HIFU pulse melted a hole into the wax coating, enabling the influx of external surrounding water through the HIFU-induced cavity. As a result, the lyophilised GBCA was hydrated, and dissolved T1-hyperintense GBCA flowed into the surrounding medium. Visual control of the three separately exposed capsules revealed a clear opening on one lateral side resembling a focal point of ultrasound energy deposition.

## Discussion

In this study, we prepared a novel thermoresponsive wax-coated capsule and provided the proof-of-concept of triggering the release of its content using an MRI-guided HIFU pulse. This drug delivery system remained intact in simulated body fluids at physiological temperature and released its cargo very rapidly when a 200-W HIFU pulse was applied. The wax-coated capsule and energy exposure were tracked using a T2-weighted MRI sequence, and the release was monitored with a T1-weighted MRI sequence based on an encapsulated lyophilised GBCA, which becomes T1-hyperintense upon hydration. We therefore provide an externally triggerable drug delivery system for locally and temporally supertargeted drug release to address an unmet clinical need for personalised local therapy in the GI tract.

The thermoresponsive properties of the wax coating were evidenced in a dissolution test where non-coated capsules released the barium sulphate after few minutes at 37 °C, while the wax-coated capsules disintegrated only after the temperature had been increased to 43 °C (*n* = 3, Fig. 3c). With regard to the complete disintegration of the capsule, these findings are most likely translatable to any cargo if the drug is sufficiently water-soluble. We further examined the release kinetics of an uncoated capsule containing lyophilised gadoterate on T1-weighted MRI. Upon incubation in water, the uncoated capsules were initially visible due to their T1-hypointense signal (*n* = 3, Fig. 3d) and gradually turned the surrounding medium into a T1-hyperintense environment (Additional file 2: Movie S1). The water-driven disintegration of the uncoated capsule allowed the hydration and release of the encapsulated T1 contrast agent which subsequently diffused slowly into the surrounding medium, rendering it T1-hyperintense (Fig. 3e). Dynamic T1-weighted imaging revealed that without any external energy deposition the release of the uncoated capsule content started at 8.3 ± 0.6 min (*n* = 3, Additional file 2: Movie S1) after water exposition.

To show the high resistance of the wax-coated capsules, they were exposed to simulated GI fluids. The harsh conditions in gastric and intestinal fluid USP stem from the high enzymatic activity (pepsin and protease-, amylase-, lipase-containing pancreatin), the extremely low pH of 1.2 in simulated gastric fluid, and the steep pH increase to 6.8 upon incubation in simulated intestinal fluid [16, 34]. The wax-coated capsules remained intact under all these conditions without any significant change in weight (*p* = 0.400, *n* = 3, Fig. 4d), which underlines the high resistance of the wax coating in the tested conditions. Other proposed HIFU-triggered drug delivery systems such as lipid vesicles [19] would probably not resist the harsh conditions of the GI tract, as they are destabilised by bile salts [12].

To demonstrate the release of the wax-coated capsule by a HIFU pulse, we embedded the wax-coated capsule in a HIFU phantom. The wax-coated capsule was identified as a hypointense signal both on T2- and T1-weighted MRI which is most probably related to the diamagnetic properties of the encapsulated barium and the low amount of water protons in the coating and capsule interior (Fig. 5a, b) [40]. The wax coating seemed to be of minor importance for the signal intensity, as fat leads to hyperintensity both on T1- and T2-weighted MRI [41]. Being surrounded by the bright water signal, the wax-coated capsule was particularly well visible on T2-weighted MRI (Fig. 5a), most likely the preferred modality to locate the capsule *in vivo* (*e.g.* in the GI tract). After the targeted application of a 200 W HIFU pulse, which is commonly used in clinical practice [42–44], a hyperintense signal was observed on T1-weighted MRI (Fig. 5d) close to the maximum energy exposure site. The HIFU pulse had most probably induced the disintegration of the wax coating by melting a lateral cavity into the wax coating, leading to the dissolution of the capsule shell and a subsequent influx of the surrounding water into the capsule core, hydrating the lyophilised GBCA which resulted in a T1-hyperintense signal. The wax coating was therefore disintegrated selectively and very rapidly by a HIFU pulse, and the release of the cargo monitored on T1-weighted MRI.

Furthermore, the Michelangelo method, in which the capsules are casted in the coating liquid and subsequently carved out in the desired dimensions, provided capsules of similar dimensions and weight with a relative standard deviation of < 3% (*n* = 6, Fig. 3b). This versatile coating method promises to be especially useful for waxy and highly viscous coating substrates, for which dip- or spray-coating methods can be challenging. Recently, a temperature-sensitive capsule with an eicosane- and superparamagnetic iron oxide-containing coating was reported which was based on a dip-coating method [45]. This system was able to release its content upon applying a magnetic field which increased the temperature in the coating and led to the dissolution of the capsule. In comparison, our system comprises several advantages: the potentially better controllable and targeted temperature increase provided by the external heat source, the possibility to verify the drug release due to the encapsulated lyophilised but soluble contrast agent, the higher and more suitable melting point of 43 °C as compared to 36.5 °C for eicosane (*i.e.* lowering the risk of inadvertent release in tumour- or inflammation-induced fever [46]), the opportunity to co-localise the capsule using computed tomography or X-ray based on the encapsulated radiopaque barium sulphate, and the food excipient status of lanolin and cetyl alcohol (in contrast to eicosane which is not monographed in the USP).

Admittedly, our study bears several limitations. We could not test the HIFU-triggered release *in vivo* due to ethical considerations (*i.e.* absent Food and Drug Administration clearance for use in the GI tract), institutional limitations concerning large animal experiments, and the inaccessibility of an MR-guided HIFU for rodents. Indeed, small injuries (*e.g.* burns) might be caused by the HIFU pulse [47], even though our chosen model is far from ablation effects described at higher temperatures. Limitations considering a translation to the *in vivo* setting include the risk of GI perforation by the HIFU beam and intraluminal gas which could interfere with the HIFU pulse. To address the risk of interference by intraluminal air on the HIFU beam, we propose fluid substitution (*e.g.* water or hydrogel) in the proximal or distal GI tract, and the use of osmotic (*e.g.* poly(ethylene glycol)) or contact laxatives (*e.g.* bisacodyl) [48, 49]. Administering laxative agents would bear the additional advantage of increasing the background signal on T2-weighted MRI and the distention of the GI tract, both of which would facilitate the localisation of the T2-hypointense wax-coated capsule [50]. To mitigate the risk of bowel wall perforation, reducing the movements of the bowel (*e.g.* with intravenous glucagon and/or intramuscular hyoscine [51]) would be a promising strategy, as the HIFU beam shows very high accuracy and precision in aperistaltic organs [52]. The risk could additionally be minimised by employing a wax mixture with a higher content of lanolin which would necessitate even lower temperature increases to release the cargo than the one used in our study (Table 1). The risks related to bowel wall perforation could further be lowered by replacing the filling agent barium sulphate with safer alternatives such as contrast agents permitting visualisation using different imaging methods (single-photon emission computed tomography, positron emission tomography, ultrasound, etc.) or inert substances (*e.g.* glucose). While we cannot fully exclude the formation of degradation products in the hydroxypropyl methylcellulose capsules upon heating to 43 °C, we hypothesise this risk to be very low with regard to the much higher temperatures employed in other formulation procedures of this excipient (*e.g.* hot-melt extrusion, film coating of tablets) [30, 53, 54]. Similar considerations apply for the melting of the lanolin/cetyl alcohol mixture, which is most likely associated with low chemical degradation with regard to the temperatures employed in other formulation procedures of these excipients (*e.g.* suppository preparation, emulsification) [30]. Moreover, we cannot fully exclude a partial release of the capsule cargo based on the MRI data with regard to potential differences in the dissolution kinetics. Quantification of the contrast release was not possible with regard to the current set-up [55]. We therefore advise to determine the release kinetics for any incorporated drug separately, ideally using sink conditions and biorelevant media [56–58]. Furthermore, the simulated body fluids employed in our release testing are an *in vitro* system, which is not identical with the complexity encountered *in vivo* [16, 34, 35]. By choosing simulated GI fluids described in the USP, however, we believe to have approximated the harshest conditions available in the human body [16, 34, 35]. Finally, the HIFU-MRI hybrid system used in our study has still limited reproducibility in achieving the targeted temperature at the desired location.

In the local treatment of inflammatory bowel diseases and GI tumours, our wax-coated capsule platform may address the currently unmet clinical need for a highly localised and time-specific rapid drug release. The spatial precision of our system cannot be reached with currently available dosage forms, which generally rely on pH changes. With regard to the high loading capacity of our macroscopic capsule-based system, the high biocompatibility of the excipients, and the combination with MRI-guided HIFU allowing a highly precise and rapid temperature increase as well as MRI-guided localisation of the capsule and verification of the release of its cargo, our system is a truly unique platform for supertargeted drug release in the GI tract. An externally triggered drug delivery system would enable an unconstrained selection of the site and kinetics of the drug action and may be combined synergistically with the therapeutic action of the heat source (*e.g.* thermoablation) [19, 29]. Micronising the wax-coated capsule system could provide the opportunity of extragastrointestinal administration such as in solid tumours (*e.g.* breast and prostate cancer). Promising approaches include a local deposition prior to neoplastic resection to decrease the tumour burden or during resection such that a chemotherapeutic drug could be released later (*e.g.* to treat a local recurrence). Naturally, a more thorough safety study would be required for a systemic exposure to the wax. Furthermore, the sequential release of drugs from capsules with different melting temperatures promises to create new opportunities for combination therapy and sophisticated sequential (*i.e.* pulsatile) release profiles. In addition to the actively induced temperature increase, passive triggers (*i.e.* physiological temperature increases such as hyperthermia or fever) may conceptually be employed as well if a wax mixture with an appropriate melting point is chosen.

In conclusion, we reported a reproducible method to coat a capsule with a thermoresponsive wax coating, the proof-of-concept of using an MRI-guided HIFU pulse to actively trigger the release of the wax-coated capsule, and a method to monitor the release based on the hydration of a lyophilised contrast agent. The wax-coated capsule platform therefore provides the opportunity for temporally and spatially supertargeted drug release and promises to address an unmet clinical need for personalised local therapy.

## Additional files

Additional file 1: **Tables S1** and **S2.** Quality control of lanolin and cetyl alcohol. (DOCX 208 kb)

Additional file 2: **Movie S1.** Disintegration of uncoated lyophilized gadoterate-containing capsule in water at room temperature followed by dynamic T1-weighted MRI (1.5 T). (WMV 801 kb)

## Abbreviations

CT: Computed tomography
GBCA: Gadolinium-based contrast agent
GI: Gastrointestinal
HIFU: High-intensity focused ultrasound
MRI: Magnetic resonance imaging
USP: United States Pharmacopoeia
