# Supplementary material for: An MRI-guided HIFU-triggered wax-coated capsule for supertargeted drug release: a proof-of-concept study
Source: Eur Radiol Exp. 2019 Mar 5;3:11. doi: 10.1186/s41747-019-0090-9 (PMC6401064; doi:10.1186/s41747-019-0090-9)

**Additional file 1**

**An MRI-guided HIFU-triggered wax-coated capsule for supertargeted drug release**

**Original Research Article**

**Table S1.** Quality control of lanolin (source Hänseler AG, Switzerland).


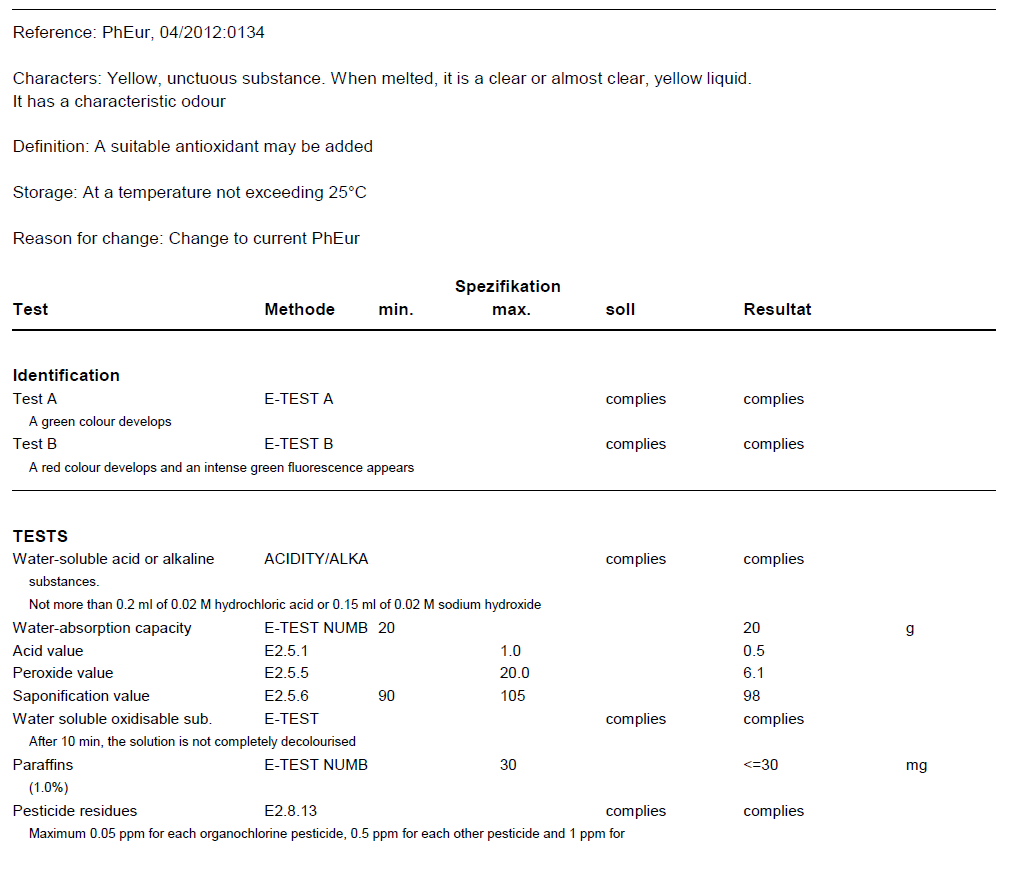


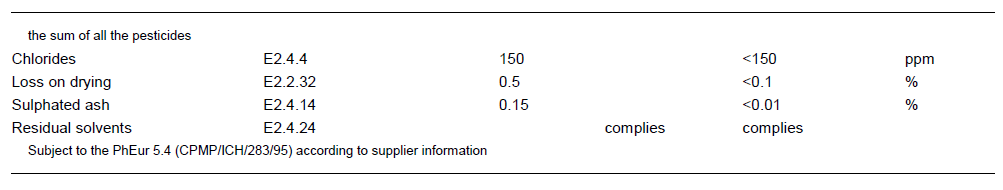


**Table S2.** Quality control of cetyl alcohol (source Hänseler AG, Switzerland).


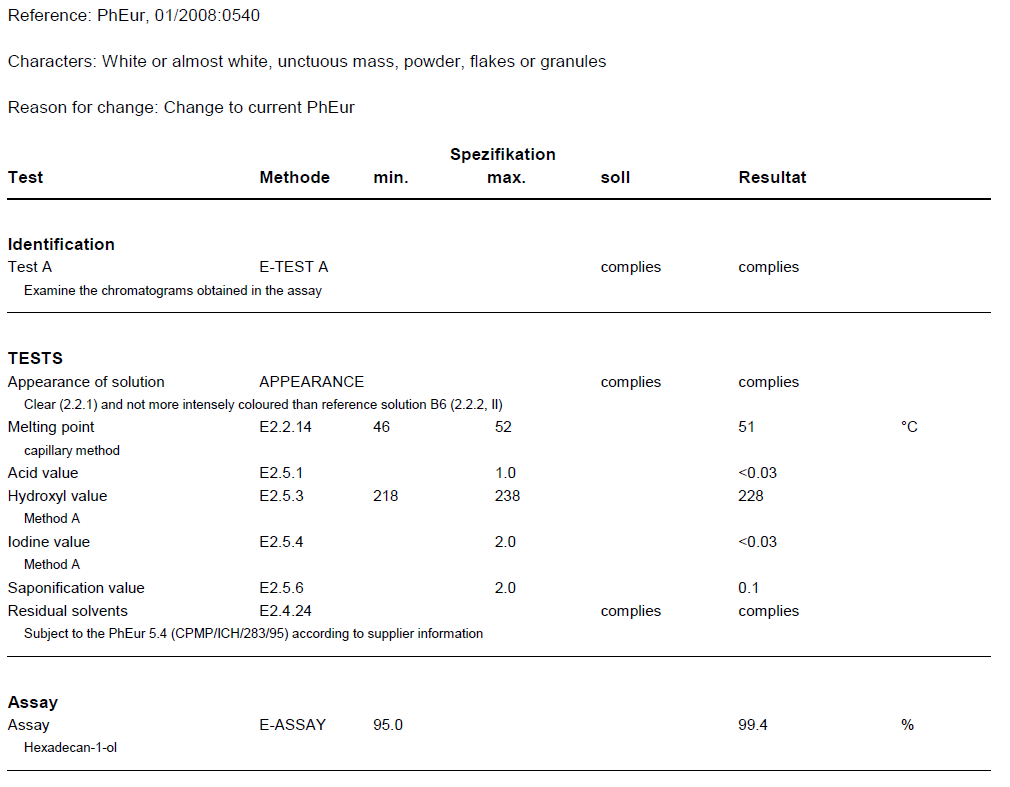

Supplement: Supplementary file 1 — Tables S1 and S2. Quality control of lanolin and cetyl alcohol. (DOCX 208 kb) [file 41747_2019_90_MOESM1_ESM.docx]
